# Changes in the Mental Health of Children and Adolescents during the COVID-19 Lockdown: Associated Factors and Life Conditions

**DOI:** 10.3390/ijerph19074120

**Published:** 2022-03-30

**Authors:** Rosa Bosch, Mireia Pagerols, Raquel Prat, Gemma Español-Martín, Cristina Rivas, Montserrat Dolz, Josep Maria Haro, Josep Antoni Ramos-Quiroga, Marta Ribasés, Miquel Casas

**Affiliations:** 1 SJD MIND Schools Program, Hospital Sant Joan de Déu, Institut de Recerca Sant Joan de Déu, 08950 Barcelona, Spain; mireia.pagerols@sjd.es (M.P.); raquel.prat@sjd.es (R.P.); cristina.rivas@sjd.es (C.R.); miquel.casas@sjd.es (M.C.); 2 Departament de Psiquiatria i Medicina Legal, Universitat Autònoma de Barcelona, 08193 Bellaterra, Spain; gespanol@vhebron.net (G.E.-M.); jaramos@vhebron.net (J.A.R.-Q.); 3 Centro de Investigación Biomédica en Red de Salud Mental (CIBERSAM), Instituto de Salud Carlos III, 28029 Madrid, Spain; montserrat.dolz@sjd.es (M.D.); jmharo@pssjd.org (J.M.H.); marta.ribases@vhir.org (M.R.); 4 Centre for Health and Social Care Research (CEES), University of Vic-Central University of Catalonia (UVic-UCC), 08500 Vic, Spain; 5 Servei de Psiquiatria, Vall d’Hebron Hospital Universitari, 08035 Barcelona, Spain; 6 Grup de Psiquiatria, Salut Mental i Addiccions, Vall d’Hebron Institut de Recerca (VHIR), Vall d’Hebron Hospital Universitari, 08035 Barcelona, Spain; 7 Child and Adolescent Mental Health Research Group, Institut de Recerca Sant Joan de Déu, 08950 Barcelona, Spain; 8 Child and Adolescent Psychiatry and Psychology Department, Hospital Sant Joan de Déu, 08950 Barcelona, Spain; 9 Research and Developmental Unit, Parc Sanitari Sant Joan de Déu, 08830 Sant Boi de Llobregat, Spain; 10 Department of Genetics, Microbiology and Statistics, Faculty of Biology, Universitat de Barcelona, 08028 Barcelona, Spain

**Keywords:** COVID-19, lockdown, life conditions, mental health problems, children, adolescents

## Abstract

This study investigated the psychological impact of the coronavirus disease 2019 (COVID-19) among youth by analyzing their emotional/behavioral problems before and during the long-lasting lockdown in Spain. For that purpose, 699 parents with children aged 6–17 and 552 adolescents aged 12–17, who completed the parent and adolescent version of the Strengths and Difficulties Questionnaire at the beginning of 2019, responded to a survey from 26 May to 15 June 2020 that assessed psychological well-being and life conditions during quarantine (i.e., sociodemographic characteristics, situation before the lockdown, physical environment and accompaniment during the lockdown, COVID-related variables). According to both parent- and self-reports, children and youth experienced a significant worsening in emotional symptoms, conduct problems, hyperactivity/inattention, peer problems, and total difficulties subscales. Findings also suggested that impairment was mainly associated with variables related to the child’s situation prior to home quarantine, the quality and quantity of the child’s social networks during the lockdown, the daily routines the child followed, the concerns the child had about health, and the presence of economic and learning problems caused by the COVID-19. Thus, the present investigation emphasizes the need for carefully monitoring the mental health of younger people, provides guidance for the development of interventions that mitigate some of the psychological difficulties faced in a situation of confinement, and highlights the importance of paying special attention to high-risk groups.

## 1. Introduction

On 11 March 2020, the World Health Organization announced that the novel coronavirus disease 2019 (COVID-19), which emerged from Wuhan, China, in December 2019, had reached pandemic status, and suggested a series of disease containment strategies to curb the spread of the infection, such as physical distancing and lockdowns. Specifically, Spain was one of the most affected countries in Europe during the first COVID-19 outbreak, with stringent restrictions on movements, school closures, and stay-at-home orders from 14 March, when the Spanish government declared the emergency situation, to June 2020. Hence, face-to-face education was suspended, extracurricular and outdoor leisure activities were canceled, and social contacts were restrained, while exposure to the internet and social media increased. The disruptions caused by these measures in daily routines, structure, and quantity of social connections are likely to have a substantial effect on people’s psychological functioning. In particular, they might be especially profound for adolescents, as social environment and peer interactions are becoming increasingly important in brain development, self-concept construction, and mental well-being during this age [1]. Indeed, adolescents’ participation in community activities has been linked to positive outcomes, such as improved academic achievement, higher levels of optimism and satisfaction with life, self-esteem, and resiliency [2,3,4]. Loades et al. [5], on the other hand, recently conducted a rapid systematic review and found that social isolation and loneliness increased the risk for depression and anxiety in healthy young people. Of note, the only included study that investigated the psychological responses of children to pandemic disasters reported higher levels of posttraumatic stress after enforced isolation and quarantine [5,6].

Adolescence also represents a period of heightened vulnerability to mental health problems because of the lack of proper emotional regulations and coping strategies [7,8]. As a result, youth exposed to natural catastrophes (e.g., hurricanes, earthquakes) might suffer from greater stress and affective conditions [9]. Notably, the rapid expansion of the initial outbreak across the world and the implementation of unprecedented mitigation measures make COVID-19 a unique and potentially more threatening experience in comparison to previous crises [10]. In this sense, a preliminary report conducted in China during the second week of February 2020 showed that children and adolescents commonly exhibited emotional and behavioral problems, such as clinginess, inattention, and irritability [11]. Symptoms of depression, anxiety, and posttraumatic stress disorder were also highly prevalent among young people [12,13,14,15] and were later reported in studies from other geographical areas [8,16]. Interestingly, researchers have also assessed the effects of home quarantine on the mental health of children and adolescents, such as Orgilés et al. [17], who determined for the first time in Western countries how the COVID-19 lockdown affected the well-being of children from Italy and Spain aged 3 to 18 years based on parents’ perceptions. Results indicated that the most frequent symptoms were difficulty concentrating, boredom, irritability, restlessness, nervousness, feelings of loneliness, uneasiness, and worries [17]. Consistently, conduct, peer, and total problems, as evaluated by 197 parents through the Strengths and Difficulties Questionnaire (SDQ), increased after lockdown in an independent sample of 13-year-old adolescents from Barcelona, Spain, although hyperactivity/inattention remained stable and emotional problems decreased [18].

Moreover, Ezpeleta et al. [18] examined the life conditions of youth during the lockdown and identified that their psychological well-being was mostly related to the activities that they kept up with, their concerns about health, the behavior of adults, the discussions in the family, and the quality of their relationships with others. In this vein, digital applications (i.e., social media, video chatting, blogging, online gaming), which allow communication and socialization, might mitigate the potentially negative effects of physical distancing in young people [1,19]. Nevertheless, excessive consumption of digital entertainment, particularly online gaming, can also be harmful [20], especially when it displaces other daily activities, such as personal care, exercise, or social functioning. Indeed, previous investigations showed that greater amounts of time spent on video games are associated with physical conditions (e.g., obesity, repetitive strain disorders), mental health problems (e.g., inattention, depression, anxiety) and poorer academic performance, in addition to acting as risk factor for the development of pathological and addictive behaviors [21,22,23].

Further risk factors for psychological distress found in children and adolescents include gender, age, place of residence, low socioeconomic status, migration background, and the number of COVID-related hardships that the family experiences (e.g., job loss, income loss, illness) [15,24,25]. Accordingly, Xie et al. [26] previously reported that Chinese students from the second to the sixth grades, who were more worried about being affected by the epidemic and less optimistic were at a greater risk for depressive symptoms after 34 days of COVID-19 home confinement. Other studies, on the other hand, have suggested that optimal lockdown life conditions (e.g., sufficient space at home, presence of a garden), enough sleep, moderate physical exercise, and participation in distance learning might be protective factors for anxiety and hyperactivity [14,27].

Most research, however, has focused on healthy populations, although children and adolescents with preexisting mental conditions might have more difficulty adjusting to lockdowns [28,29] given the uncertainty, loss of daily routines, and disruptions to support services caused by the COVID-19 restrictions [30]. The first study investigating the mental health of children with attention-deficit/hyperactivity disorder (ADHD) during the COVID-19 outbreak showed that their ADHD symptoms significantly worsened compared to normal state [31]. Similarly, Nonweiler et al. [32] observed a higher prevalence of emotional and conduct problems, and fewer prosocial behaviors among children and adolescents with neurodevelopmental disorders (i.e., autism spectrum disorder, ADHD) in a cross-sectional United Kingdom parent-reported survey. Nevertheless, contradictory results have also been found, since research conducted in France revealed that most children and youth with ADHD experienced stability or improvement in well-being and anxiety, which, according to parents, was attributed to less school-related strain, flexible organization, lower exposure to negative feedback, and increased awareness of their child’s difficulties [27].

Furthermore, these investigations are limited in their ability to inform how much children’s emotional and behavioral well-being has changed due to the home quarantine imposed by COVID-19, since information on the mental health status before the lockdown was unavailable, collected retrospectively, or came from a different sample. To the best of our knowledge, there is only one study in which 675 first-year college students completed a full assessment of behavioral and emotional functioning at the beginning and at the end of the spring semester 2020 to test the effects of the COVID-19 pandemic and the resulting mitigation strategies on mental health and wellness [10]. Findings from this report evidenced a modest but persistent negative impact on students’ externalizing symptoms, attention problems, mood and wellness behaviors, especially for those who perceived the crisis as more personally disruptive [10].

Given that Spain had one of the most restrictive and prolonged home confinements, the present study examined the psychological impact of the COVID-19 lockdown, while addressing some of the limitations in previous investigations by collecting data on mental health from the same individuals at two time intervals and through different informants. Specifically, we conducted a cross-sectional survey from 26 May to 15 June 2020 involving Spanish children and adolescents, who were evaluated for emotional/behavioral problems before the beginning of quarantine through the parent- and self-reported version of the SDQ, to analyze changes in their mental health during the lockdown. In addition to control for previous measures of psychopathology, we explored multiple factors that might mitigate or contribute to these effects, including the child’s mood and behavior before the lockdown, preexisting mental conditions (e.g., neurodevelopmental disorders), and their life conditions during the lockdown. Finally, the large size and age range of our sample also surpass earlier studies.

Our main hypotheses were as follows:

1. The COVID-19 lockdown would affect the mental health of children and adolescents from Spain. In particular, we assumed that they would show significantly higher emotional and behavioral problems during home quarantine than before the COVID-19 outbreak.
2. The mental health impairment experienced by children and adolescents would be associated with their sociodemographic characteristics, situation prior to home quarantine, physical environment and accompaniment during the lockdown, and COVID-related variables (e.g., concerns about health, presence of learning problems and economic difficulties in the family caused by the pandemic). For instance, we expected that those with preexisting mental conditions, such as emotional/behavioral problems and neurodevelopmental disorders, or limited housing facilities would be particularly vulnerable to the potentially negative effects of quarantine. In contrast, maintaining social interaction, albeit remotely, with family members, friends, classmates, and teachers, as well as daily life routines, would be protective factors.

## 2. Materials and Methods

### 2.1. Participants and Procedure

**Figure 1:**
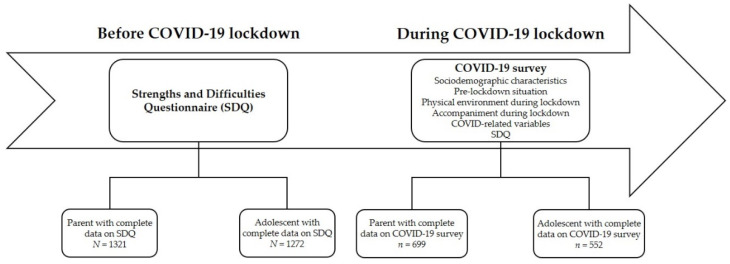
Diagram of the study design process.

This study is a subsample of a larger, ongoing research called INSchool, which started in 2011 with the aim of identifying the mental health problems of children and adolescents in a school setting. A detailed description of the study design and methodology is provided elsewhere [33,34]. Specifically, 1321 families of students aged 6 to 17, who had been evaluated at the beginning of 2019 with the parent SDQ as part of the INSchool research, were invited to complete a survey that assessed the psychological well-being and life conditions of their children during the home quarantine imposed due to COVID-19. In addition, we asked students in secondary education (*N* = 1272), who had also completed the adolescent SDQ at the beginning of 2019, to fill in a self-reported version of this form. Data were collected from 26 May to 15 June 2020, using a questionnaire created with an online platform and distributed to all participants through email. In total, 699 parents with children aged 6 to 17 (participation rate = 52.9%) and 552 adolescents aged 12 to 17 (participation rate = 43.4%) fulfilled the survey and were included in the analysis (Figure 1). Information from both sources was available for 216 of the analyzed youth.

Participants, compared to non-participants, were younger (parent-reported sample: 10.61 vs. 11.45 years, *p* < 0.001; adolescent-reported sample: 12.91 vs. 13.00 years, *p* = 0.005), predominantly from public schools (parent-reported sample: 73.5% vs. 60.9%, *p* < 0.001; adolescent-reported sample: 61.6% vs. 51.9%, *p* = 0.001), and reported higher prosocial behavior (parent-reported sample: 7.85 vs. 7.59, *p* = 0.026; adolescent-reported sample: 7.97 vs. 7.53, *p* < 0.001) and lower levels of conduct problems (parent-reported sample: 1.27 vs. 1.48, *p* = 0.015; adolescent-reported sample: 1.51 vs. 1.89, *p* < 0.001), peer problems (parent-reported sample: 1.14 vs. 1.53, *p* < 0.001; adolescent-reported sample: 1.28 vs. 1.46, *p* = 0.024) and total difficulties (parent-reported sample: 7.06 vs. 7.82, *p* = 0.016; adolescent-reported sample: 8.63 vs. 9.42, *p* = 0.003) before the COVID-19 outbreak. Additional differences were found in the adolescent-reported sample, with participants being more frequently girls (61.6% vs. 42.9%, *p* < 0.001) and exhibiting less symptoms of hyperactivity/inattention at the beginning of 2019 (3.42 vs. 3.84, *p* = 0.001).

### 2.2. Measures

The online survey distributed during home quarantine was developed by our research team and gathered information from two different informant perspectives (i.e., parents and adolescents) through 68 and 65 items, respectively, on: (a) sociodemographic characteristics; (b) the child’s situation before the lockdown; (c) physical environment during the lockdown; (d) accompaniment during the lockdown; (e) COVID-related variables; and (f) the mental health of children and adolescents during the lockdown, as evaluated through the SDQ.

Sociodemographic variables included the child’s gender, age, and the type of school.

Pre-lockdown situation variables included questions regarding family environment (e.g., ‘How was the family environment before lockdown?’), child’s mood and behavior (e.g., ‘How was your child’s/your behavior before lockdown?’), presence of neurodevelopmental disorders (e.g., ‘Does your child have a diagnosis of a neurodevelopmental disorder, such as ADHD, learning disorder, or autism spectrum disorder?’, ‘Did your child receive any medication before lockdown?’), and use of video games prior to home quarantine (e.g., ‘Did your child/you play videogames before lockdown?’).

Physical environment during lockdown variables included questions referring to the child’s city of residence, type and size of the child’s house, availability of outdoor areas, and adequate facilities to follow virtual learning. These items were summarized in two variables created ad hoc: comfort home, ranging from 2 to 11, based on the type of housing (1 = apartment, 2 = house), size (1 ≤ 70 m^2^, 2 = 70–89 m^2^, 3 = 90–120 m^2^, 4 ≥ 120 m^2^), availability of outdoor areas (0 = none, 1 = balcony, 2 = yard, 3 = terrace, 4 = garden), and brightness (0 = no, 1 = yes), and study facilities, ranging from 1 to 7, based on the availability (0 = no, 1 = yes) of an internet connection at home, a place to study, computer, own cell phone, tablet, own desk, and own bedroom. Higher scores reflected higher comfort and better study facilities, respectively.

Accompaniment during lockdown variables included questions about family environment (e.g., ‘How has the family environment been during lockdown?’, ‘Did your family/you enjoy the time spent together/with your family during lockdown?’), social distancing from relatives (e.g., ‘Has any relative been far from home because of the lockdown?’), and people sharing the same house during lockdown (e.g., ‘With who has/have your child/you lived during lockdown?’). Respondents were also asked about the use of social media to stay in touch with teachers (e.g., ‘Has/Have your child/you followed online classes with his or her/your teachers during lockdown?’), friends (e.g., ‘During the lockdown period, has/have your child/you stayed in touch with friends through online communication systems?’), and family members who lived apart (e.g., ‘During the lockdown period, has/have your child/you stayed in touch with relatives who lived apart through online communication systems?’).

COVID-related variables included the presence of economic problems in the family caused by the pandemic (e.g., ‘Has your household income declined due to the pandemic?’, ‘Has your family had economic problems due to the pandemic?’), concerns about health (e.g., ‘Have you been concerned about your health or your relatives’ health during lockdown?’), questions regarding whether children contracted COVID-19 or knew someone who became ill or died because of the virus (e.g., ‘Has/Have your child/you contracted COVID-19?’, ‘Has/Have your child/you been hospitalized for COVID-19?’, ‘Has any of your child’s/your relatives died from COVID-19?’), and the respondent’s perception on how quarantine affected learning and daily routines, such as diet, sleep, study, physical exercise, and screen time (e.g., ‘Has/Have your child/you practiced physical activity during lockdown?’, ‘Do you think your child’s/your learning has been delayed by the lockdown?’, ‘Have your child/you played video games during lockdown?’).

The mental health of children and adolescents during the lockdown was measured with the SDQ [35], a standardized screening instrument that had already been administered at the beginning of 2019, before the onset of the COVID-19 crisis. The questionnaire covers common areas of social, emotional, and behavioral functioning through 25 items distributed across five subscales: emotional symptoms, conduct problems, hyperactivity/inattention, peer problems, and prosocial behavior. Further, a total difficulties score may be derived by summing the first four subscales. The parent and adolescent versions of the Spanish SDQ used in the present study have been validated as feasible instruments to identify mental health problems in children aged 5 to 17 [34]. Ordinal alpha coefficients yielded generally adequate reliability estimates across all subscales, both before (parent version, range = 0.78–0.91; adolescent version, range = 0.70–0.88) and during the lockdown (parent version, range = 0.71–0.88; adolescent version, range = 0.69–0.89). The frequency of subjects with a score in the clinical range on each SDQ subscale was estimated based on the bands developed by Español-Martín et al. [34], who provided Spanish normative data according to the child’s gender, age, and type of informant.

The full text of the online surveys, including all questions and response options, is available as an online resource (Tables S1 and S2).

### 2.3. Statistical Analyses

All analyses were performed with SPSS 25.0. (IBM) Descriptive statistics were used to illustrate the sociodemographic and other selected characteristics of the samples. According to the Kolmogorov–Smirnov test (*p* < 0.05), SDQ scores were not normally distributed. Thus, changes across the subscales and total difficulties scores before and during COVID-19 lockdown were analyzed with the Wilcoxon signed rank test for matched pairs. As suggested by Tomczak and Tomczak [36] for non-parametric tests, the correlation coefficient *r* was calculated to estimate effect sizes, which were interpreted as small for *r* values < 0.30, moderate for values ≥ 0.30, and large for values ≥ 0.50 [37]. For each significant SDQ subscale, the pre-lockdown score was subsequently subtracted from that during the lockdown to explore through univariate analyses the associations between changes in mental health problems and each of the items included in the sociodemographic, pre-lockdown situation, physical environment during the lockdown, accompaniment during the lockdown, and COVID-related variables sections of the surveys. Given the non-normal distribution of the variation across SDQ scores, a variable called “impaired status” was created to test in a set of multivariate logistic regression models those items found to be statistically significant after adjusting for the number of significant SDQ subscales in the parent (*p* ≤ 0.013 (i.e., 0.05/4)) and adolescent-reported (*p* ≤ 0.010 (i.e., 0.05/4)) samples. Individuals with a zero or negative result in any of the SDQ subscales, except for the prosocial behavior, were considered non-impaired, while those with positive values were classified as impaired. A two-sided *p* value of 0.05 was set as the significance level in the multivariate regression analyses.

## 3. Results

### 3.1. Sample Characteristics

The parent-reported sample was composed of 362 (51.8%) boys and 337 (48.2%) girls (*M* = 12.5 years; *SD* = 3.19), while the adolescent-reported sample was composed of 217 (39.3%) boys and 335 (60.7%) girls (*M* = 14.8 years; *SD* = 0.62). Most of the students attended public schools (parent-reported sample: 73.1%; adolescent-reported sample: 61.8%) and had been quarantined at home for approximately 79 days (parent-reported sample: *M* = 78.8, *SD* = 5.12; adolescent-reported sample: *M* = 78.7, *SD* = 5.29).

At the beginning of 2019, before the COVID-19 outbreak, the proportion of children who fell within the clinical range on each SDQ subscale was as follows, according to parents’ perceptions: emotional symptoms, 12.9%; conduct problems, 11.7%, hyperactivity/inattention, 14.2%; peer problems, 13.4%; prosocial behavior, 17.2%; and total difficulties, 8.87%. During home quarantine, the prevalence of clinically relevant symptoms rose to 19.3% for emotional symptoms, 18.9% for conduct problems, 22.6% for hyperactivity/inattention, 21.0% for peer problems, 14.6% for prosocial behavior, and 18.7% for total difficulties. Similarly, self-reports indicated that the percentage of adolescents who scored above the clinical cutoff before and during the COVID-19 lockdown increased from 10.9% to 18.3% for emotional symptoms, 16.5% to 23.2% for conduct problems, 10.1% to 16.1% for hyperactivity/inattention, 18.7% to 27.4% for peer problems, 13.6% to 16.8% for prosocial behavior, and 9.96% to 17.0% for total difficulties.

The family environment and child’s behavior prior to home quarantine was good or very good, according to the vast majority of parents (91.7% and 85.6%, respectively). Almost 58.0% also reported that their children used to play video games and 23.5% confirmed the presence of a neurodevelopmental disorder. On the other hand, the proportion of adolescents who perceived their behavior and family environment as good or very good was slightly lower (73.4% and 78.2%, respectively), and less than half (49.3%) reported playing video games before the COVID-19 pandemic. Regarding the physical environment and accompaniment during the lockdown, most of the participants stayed in apartments (parent-reported sample: 60.2%; adolescent-reported sample: 62.7%) with sizes larger than 90 m^2^ and share the house with both parents (parent-reported sample: 86.7%; adolescent-reported sample: 85.9%) and one sibling (parent-reported sample: 58.9%; adolescent-reported sample: 52.4%). Around 30.0% of respondents reported financial difficulties caused by the pandemic (parent-reported sample: 36.1%; adolescent-reported sample: 24.6%), had moderate health concerns (parent-reported sample: 25.6%; adolescent-reported sample: 30.1%), and knew a relative or a friend who had been infected with coronavirus (parent-reported sample: 20.9%; adolescent-reported sample: 29.1%). Nevertheless, only a few children and adolescents had a confirmed diagnosis of COVID-19 (parent-reported sample: 0.85%; adolescent-reported sample: 1.63%) or knew someone who died because of the virus (parent-reported sample: 2.00%; adolescent-reported sample: 3.80%). A detailed description of the parent- and adolescent-reported samples, including sociodemographic and other selected characteristics, is provided in Table 1.

### 3.2. Changes in Psychological Symptoms before and during the COVID-19 Lockdown

Table 2 presents the parent and adolescent scores on the SDQ subscales, obtained at the beginning of 2019, before the COVID-19 outbreak, and during the home quarantine imposed by the pandemic since 14 March 2020. Children and adolescents experienced a significant worsening in subscales involving emotional symptoms, conduct problems, hyperactivity/inattention, and peer problems, according to both parent- and self-reports, although effect sizes were in the small range (*r* = 0.16–0.29). The total difficulties score also increased during the lockdown, based on parent ratings (pre-lockdown: *M* = 7.02 (*SD* = 5.57), during the lockdown: *M* = 9.40 (*SD* = 5.61), *p* < 0.001) and adolescent ratings (pre-lockdown: *M* = 8.62 (*SD* = 5.59), during the lockdown: *M* = 11.2 (*SD* = 5.62), *p* < 0.001), with moderate effect sizes (*r* = 0.36 and *r* = 0.33, respectively). However, there were differences across informants regarding the prosocial behavior subscale, since adolescents reported lower scores during the lockdown (pre-lockdown: *M* = 7.96 (*SD* = 1.71), during the lockdown: *M* = 7.81 (*SD* = 1.77), *p* = 0.046), while parents did not perceive significant changes (Table 2).

Similarly, significant changes were found in the SDQ subscales among adolescents (*n* = 216) with overlapping information from parents (emotional symptoms: *M* = 1.52 (*SD* = 1.86) vs. *M* = 1.92 (*SD* = 1.93), *p* = 0.001, *r* = 0.16; conduct problems: *M* = 0.92 (*SD* = 1.22) vs. *M* = 1.28 (*SD* = 1.28), *p* < 0.001, *r* = 0.22; hyperactivity/inattention: *M* = 2.31 (*SD* = 2.45) vs. *M* = 2.90 (*SD* = 2.55), *p* < 0.001, *r* = 0.24; peer problems: *M* = 1.03 (*SD* = 1.50) vs. *M* = 1.70 (*SD* = 1.74), *p* < 0.001, *r* = 0.32; total difficulties: *M* = 5.58 (*SD* = 5.08) vs. *M* = 7.81 (*SD* = 5.13), *p* < 0.001, *r* = 0.35) and self-reports (emotional symptoms: *M* = 2.27 (*SD* = 2.11) vs. *M* = 3.11 (*SD* = 2.17), *p* < 0.001, *r* = 0.25; conduct problems: *M* = 1.34 (*SD* = 1.41) vs. *M* = 1.81 (*SD* = 1.53), *p* < 0.001, *r* = 0.20; hyperactivity/inattention: *M* = 3.37 (*SD* = 2.40) vs. *M* = 4.22 (*SD* = 2.55), *p* < 0.001, *r* = 0.26; peer problems: *M* = 1.13 (*SD* = 1.52) vs. *M* = 1.58 (*SD* = 1.53), *p* < 0.001, *r* = 0.20; total difficulties: *M* = 8.11 (*SD* = 5.47) vs. *M* = 10.7 (*SD* = 5.29), *p* < 0.001, *r* = 0.34). Scores on the prosocial behavior subscale also worsened during the lockdown according to youth (pre-lockdown: *M* = 8.00 (*SD* = 1.78), during the lockdown: *M* = 7.71 (*SD* = 1.92), *p* = 0.005, *r* = 0.13). On the contrary, parents reported a slight but significant improvement in their offspring’s prosocial behavior (pre-lockdown: *M* = 7.87 (*SD* = 1.87), during the lockdown: *M* = 8.13 (*SD* = 1.57), *p* = 0.047), although the effect size was small (*r* = 0.096).

### 3.3. Factors Influencing Mental Health Impairment during COVID-19 Lockdown

Multivariate logistic regression analyses were performed, following univariate analyses, to determine the effects of the relevant sociodemographic, pre-lockdown situation, physical environment during the lockdown, accompaniment during the lockdown, and COVID-related variables on the impaired status of participants across the significant SDQ subscales (parent-reported sample: emotional symptoms, conduct problems, hyperactivity/inattention, and peer problems; adolescent-reported sample: emotional symptoms, conduct problems, hyperactivity/inattention, peer problems, and prosocial behavior). Those variables found to be significant in univariate analyses and subsequently included in multivariate logistic regression models are available as online resources (Tables S3 and S4).

As shown in Table 3, parent reports suggested that impairment in the emotional symptoms subscale was mainly related to the child’s mood before the lockdown; that is, whether he or she had exaggerated worries (odds ratio (*OR*) = 3.01, 95% confidence interval (*CI*) = 1.28–7.06, *p* = 0.011), woke up multiple times in the middle of the night (*OR* = 2.87, 95% *CI* = 1.16–7.11, *p* = 0.023), or felt nervousness (*OR* = 1.67, 95% *CI* = 1.01–2.75, *p* = 0.046). Additionally, students in secondary education and those who experienced an academic delay during the lockdown were more likely to be impaired (*OR* = 1.62, 95% *CI* = 1.16–2.25, *p* = 0.004 and *OR* = 1.16, 95% *CI* = 1.03–1.31, *p* = 0.018, respectively), while children who played video games prior to home quarantine had a decreased risk of emotional symptoms (*OR* = 0.59, 95% *CI* = 0.43–0.81, *p* < 0.001). Impairment in the conduct problems subscale was associated with a worse child’s behavior prior to home quarantine (*OR* = 1.84, 95% *CI* = 1.43–2.38, *p* < 0.001) and not keeping up with daily routines during the lockdown (*OR* = 1.20, 95% *CI* = 1.02–1.41, *p* = 0.027). On the other hand, younger children (*OR* = 1.06, 95% *CI* = 1.00–1.12, *p* = 0.043), boys (*OR* = 1.41, 95% *CI* = 1.04–1.92, *p* = 0.027), and students who hardly followed virtual learning during the lockdown (*OR* = 1.17, 95% *CI* = 1.01–1.83, *p* = 0.035) had an increased risk of impairment in the hyperactivity/inattention subscale. The multivariate logistic regression model also determined that subjects who felt sadness before the lockdown were more likely to experience a worsening in the peer problems subscale (*OR* = 4.16, 95% *CI* = 1.47–11.8, *p* = 0.007), while those who played video games prior to home quarantine and kept up online communication with their friends during the lockdown had less odds of being impaired (*OR* = 0.60, 95% *CI* = 0.44–0.82, *p* = 0.001 and *OR* = 0.74, 95% *CI* = 0.64–0.84, *p* < 0.001, respectively). Furthermore, playing video games before the COVID-19 outbreak and having a previous diagnosis of neurodevelopmental disorder were protective factors against mental health impairment according to the total difficulties score (*OR* = 0.65, 95% *CI* = 0.46–0.90, *p* = 0.011 and *OR* = 0.52, 95% *CI* = 0.36–0.74, *p* < 0.001, respectively).

Significant results of the multivariate logistic regression analyses based on adolescent reports are presented in Table 4. Specifically, youth who woke up multiple times in the middle of the night prior to the pandemic and who were concerned about health during home quarantine had an increased risk of impairment in the emotional symptoms subscale (*OR* = 2.11, 95% *CI* = 1.14–3.93, *p* = 0.018 and *OR* = 1.18, 95% *CI* = 1.03–1.36, *p* = 0.017, respectively). Impairment in conduct problems and hyperactivity/inattention subscales were greater among students not taking advantage of the lockdown to spend more time on their school tasks (*OR* = 1.23, 95% *CI* = 1.08–1.41, *p* = 0.004 and *OR* = 1.27, 95% *CI* = 1.10–1.47, *p* = 0.001, respectively). Moreover, adolescents with less study facilities at home were more likely to experience a worsening in the hyperactivity/inattention subscale (*OR* = 1.20, 95% *CI* = 1.06–1.36, *p* = 0.005). On the other hand, having a disorganized sleep schedule before the lockdown significantly heightened the odds of impairment in the peer problems subscale (*OR* = 3.23, 95% *CI* = 1.32–7.92, *p* = 0.010), while keeping up online communication with friends during home quarantine was a protective factor (*OR* = 0.79, 95% *CI* = 0.68–0.93, *p* = 0.005). Finally, adolescents who reported COVID-related economic problems in the family were at a higher risk of being mentally impaired according to the total difficulties score (*OR* = 1.88, 95% *CI* = 1.21–2.90, *p* = 0.005). In contrast, none of the sociodemographic, pre-lockdown situation, physical environment during the lockdown, accompaniment during the lockdown, or COVID-related variables assessed in the univariate analyses showed a significant association with impairment in the prosocial behavior subscale.

## 4. Discussion

The COVID-19 pandemic has led most countries to implement a series of disease containment strategies (e.g., population confinement, physical distancing, school closures, restrictions on movements) to curb the spread of the infection. Spain was one of the most affected countries in Europe during the first COVID-19 outbreak since the implemented measures were stricter and lasted longer than other regions. After almost 2 months of home quarantine, for instance, youth could only go outside for a justified reason, which put them at risk of experiencing negative psychological effects. The present study investigated the impact of the COVID-19 lockdown on the mental health of Spanish children and adolescents by analyzing their emotional/behavioral problems before and during confinement from different informant perspectives. Additionally, we explored multiple factors (i.e., sociodemographic characteristics, situation prior to home quarantine, physical environment during the lockdown, accompaniment during the lockdown, and COVID-related variables) that might have influenced those changes.

According to both parent- and self-reports, the emotional symptoms, conduct problems, hyperactivity/inattention, peer problems, and total difficulties were significantly higher than before the outbreak. The prosocial behavior, as evaluated by adolescents, also worsened during the lockdown period, while parents did not perceive any change. These results add to previous studies from other geographical areas, showing that young people experienced various negative responses due to the pandemic, such as clinginess, fear, inattention, anxiety, depression, and symptoms of posttraumatic stress disorder [8,16]. Moreover, our findings are in line with those from Orgilés et al. [17], who determined for the first time how the COVID-19 lockdown affected the well-being of Spanish children aged 3 to 18 years and reported poor concentration, boredom, irritability, restlessness, worries, and uneasiness among the most frequent symptoms. Interestingly, Ezpeleta et al. [18] also found a significant increase in conduct, peer, prosocial, and total problems after home quarantine, as evaluated by parents through the SDQ, although emotional symptoms decreased and hyperactivity/inattention remained stable.

On the other hand, the current research suggested that mental health status was mainly associated with variables related to the child’s situation before the lockdown, accompaniment during home quarantine, concerns about health, and changes in family income, daily routines, and learning caused by the pandemic. In particular, children with worse behavior prior to home quarantine exhibited more conduct problems. Furthermore, peer problems significantly worsened among subjects who felt sadness or had disorganized sleep schedules before the lockdown, and children who already showed anxiety-related symptoms, such as exaggerated worries, nervousness, or insomnia, were more likely to experience emotional problems (Table 3 and Table 4). These results are consistent with studies showing that sleep disturbances can predict later depression and anxiety [38,39]. Finally, we found that participants with previous neurodevelopmental disorders had a lower risk of being impaired than those who were not diagnosed, according to parents’ ratings on total difficulties. In contrast, Nonweiler et al. [32] reported an increased prevalence of emotional and conduct problems, and fewer prosocial behaviors among children and adolescents with neurodevelopmental disorders, compared to UK pre-COVID-19 norms. Of note, the SDQ measures obtained by these authors during the COVID-19 pandemic and those from before did not come from the same subjects, which might be responsible for the discrepant results. Differences in the outcome variable; that is, rates of emotional/behavioral difficulties and mental health impairment, could also explain the disparity between Nonweiler et al. [32] and our investigation. In this sense, we classified as impaired those individuals with positive values after subtracting the pre-lockdown scores from that during the lockdown. Consequently, subjects with neurodevelopmental disorders might have experienced less impairment during the lockdown as they already had higher scores on the total difficulties subscale prior to home quarantine. Interestingly, however, a study conducted in France revealed that most children and youth with ADHD showed no significant changes or improved their anxiety and self-esteem since the lockdown, which, according to parents, was attributed to less school-related strain, flexible schedules that respected their child’s rhythm, and lowered exposure to negative feedback [27].

We should mention the use of video games before the lockdown, given the protective role against impairment that we observed in terms of emotional symptoms, peer problems, and total difficulties, based on parents’ perception. These results are supported by Jiao et al. [11], who reported that media entertainment was largely used at the beginning of the pandemic as a means to relieve children’s distress and address their concerns about the negative conditions they were experiencing. Additionally, gaming demonstrated positive effects on mental health by improving cognitive stimulation and reducing depression, anxiety, and loneliness [40]. The emotional protective value of video games observed under the conditions of a pandemic-imposed isolation might be related to their potential for fulfilling basic human needs, such as competency, autonomy, and relatedness, which could not be met otherwise [41]. Nevertheless, attention must be paid to the prolonged use of video games, particularly when it displaces other daily activities, since excessive gaming can also be harmful and act as a risk factor for the development of pathological addictive behaviors. Indeed, Ezpeleta et al. [18] found that spending more time than usual on screens during the lockdown was associated with higher conduct, peer, and total problems. Similarly, a longitudinal study conducted between May 2020 and April 2021 revealed that children with higher levels of screen use had greater mental health symptoms, including depression, anxiety, irritability, inattention, hyperactivity, and conduct problems [42]. Overall, research agrees that any debate on the effects of video games cannot be addressed from a rigid dichotomous approach, according to which playing video games is good or bad. Instead, online gaming should be conceptualized as a continuum, depending on the time spent playing and the characteristics of both the individual and the game (i.e., content, context, structure, mechanics) [43,44]. In particular, the current investigation suggests that playing video games in moderation may be a helpful strategy to cope with stress, aversive emotions, and loneliness during a lockdown situation, especially those that encourage physical activity and social interactions [20]. On the other hand, setting restrictions on the time, place, and content of video games seem to be effective strategies to prevent or reduce symptoms in those children who developed addictive behaviors in this pandemic period [45]. Parents should also foster alternative avenues for social interaction, meaningful leisure activities, and a wider repertoire of interests among their offspring to maintain the right balance with offline activities in their daily routines [44,45]. Finally, it is important to ensure that young people have access to mental health resources and provide therapists with training on problematic media use to help them recover from the isolation and stress of the pandemic [42].

Moreover, it is known that depression and anxiety are more likely to occur and worsen in the absence of interpersonal communication [46]. Therefore, social media, which enables communication with those far away, could also have positive effects on the mental health of people who are quarantined by reducing their feelings of isolation, panic, and worry [47]. Indeed, our results revealed that keeping up with online communication with friends during the lockdown was associated with less peer problems, according to both parent and adolescent reports, whereas students who hardly followed virtual learning had an increased risk of hyperactivity/inattention, based on parents’ perceptions. Consistently, previous investigations have related the lack of online communication with friends to higher conduct, peer, and total problems [18]. Moreover, Zhang et al. [31] demonstrated that ADHD symptoms reduced with the longer amount of time children spent online studying while they were at home during the COVID-19 outbreak. In this vein, participating in distance learning has also been found to be associated with a decreased risk of anxiety in Chinese adolescents [14]. Thus, the present study emphasizes the importance of maintaining the structure, quality, and quantity of social networks, albeit remotely, to reduce the impact of enforced physical distancing [48].

Likewise, there is evidence that keeping up with previous routines for daily life activities (e.g., hygiene, diet, sleep, study) may mitigate some of the psychological difficulties faced in a situation of confinement, including anxiety, conduct problems, and hyperactivity/inattention [14,18]. Accordingly, we observed that subjects not following their daily routines during home quarantine were more likely to exhibit conduct problems, as evaluated by parents. Furthermore, the COVID-19 outbreak also affected children’s learning and academic progression, which could have had adverse effects on their mental health. For instance, adolescents who did not take advantage of the lockdown to spend more time on their school tasks reported increased conduct and hyperactivity/inattention problems, and those experiencing academic delays, according to their parents, were at a greater risk of emotional symptoms. Youth experiencing health concerns during quarantine were also at a risk for higher emotional difficulties, as previously demonstrated [18,26,49], and those who informed about COVID-related economic problems in the family presented more psychological distress overall, which is also consistent with evidence from the current and past pandemics [21,50,51,52].

On the contrary, sociodemographic and physical environment conditions during the lockdown did not significantly impact mental health, except for younger children, boys, and subjects with less study facilities at home, who were more likely to display hyperactivity/inattention problems. In this vein, results from a recent review on earlier pandemics did not provide strong evidence that any particular demographic variables were risk factors for poor psychological outcomes after quarantine [53]. Moreover, in line with the current research, Ezpeleta et al. [18] found that the physical environment was not associated with emotional, conduct, and total difficulties. However, this finding warrants further investigation, since other studies suggested that children living in small apartments were particularly burdened by the effects of the COVID-19 pandemic [25], while optimal lockdown life conditions (e.g., sufficient space at home, presence of a garden) might compensate for the impact of ADHD symptoms in youth with the disorder [27].

Several methodological limitations should be considered when interpreting the results. First, the cross-sectional design of the study prevents from establishing causal relations between variables or the longitudinal impact of the COVID-19 outbreak on mental health. In this vein, further research is needed as data from previous pandemics suggest that the psychological effects of home quarantine can be long lasting [53]. The samples included voluntary participants and data were collected using an online questionnaire, which might have limited its representativeness of the Spanish youth by excluding participants without internet access or the necessary computer skills, resources, and support. Moreover, subjects excluded from the analysis might have felt significantly more burdened by lockdown than participants as they already reported higher mental health problems before the onset of the COVID-19 crisis. Thus, the associations found in our study might have been attenuated and require further validation. Lastly, the socioeconomic status and ethnicity of families was not evaluated, although these conditions may render children more vulnerable during a pandemic [25]. On the other hand, one of the main strengths of the present research is that, for the first time to our knowledge, we examined the impact of home quarantine due to the COVID-19 outbreak on the mental health of children and adolescents by analyzing their emotional/behavioral problems before and during the long-lasting confinement in Spain through the parent and adolescent version of the SDQ, a standardized screening instrument. In addition to control for previous measures of psychopathology and preexisting conditions (e.g., neurodevelopmental disorders), we explored multiple factors that might influence those changes, including the child’s pre-lockdown situation and life conditions during home quarantine. Nevertheless, we cannot rule out that other individual and societal factors may have influenced the differences in mental health attributed to home quarantine, since participants were first evaluated at the beginning of 2019, a year prior to the onset of the COVID-19 crisis.

## 5. Conclusions

This study demonstrates that the COVID-19 pandemic has considerably affected the psychological functioning of younger people, with significant worsening of their emotional, conduct, hyperactivity/inattention, peer, and total problems during the lockdown. Our results also suggest that the mental health status was mainly associated with variables regarding the children’s situation prior to home quarantine, the quality and quantity of their social networks during the lockdown, the daily routines they followed, the concerns they had about health, and the presence of economic and learning problems caused by COVID-19. Taken together, the present investigation emphasizes the need to carefully monitor the mental health of children and adolescents, provides guidance for the development of interventions that mitigate the psychological difficulties faced in a situation of confinement, and highlights the importance of paying special attention to high-risk groups. In this sense, clear and age-appropriate information about the disease may reduce the fears and uncertainty of subjects with anxiety-related symptoms or those who are more concerned about health. Moreover, maintaining social interaction, albeit remotely, with family members, friends, classmates, and teachers, as well as daily life routines, seem essential to reduce the impact of enforced physical distancing.

## Figures and Tables

**Table 1:** Characteristics, life conditions, and COVID-related variables of the study samples.

	Parent-Reported Sample (*N* = 699)	Adolescent-Reported Sample (*N* = 552)
**Sociodemographic characteristics**		
Gender, *n* (%)		
Male	362 (51.8)	217 (39.3)
Female	337 (48.2)	335 (60.7)
Age, *M* (*SD*)	12.5 (3.19)	14.8 (0.62)
Type of school, *n* (%)		
Public	511 (73.1)	341 (61.8)
Private	188 (26.9)	211 (38.2)
Educational stage, *n* (%)		
Primary	245 (35.1)	―
Secondary	454 (64.9)	552 (100.0)
**Pre-lockdown situation**		
Family environment, *n* (%)		
Bad or very bad	3 (0.43)	12 (2.17)
Average	55 (7.89)	108 (19.6)
Very good or good	641 (91.7)	432 (78.2)
Child’s mood, *n* (%)		
Insomnia	21 (3.00)	62 (11.2)
Hypersomnia	12 (1.72)	24 (4.35)
Waking up multiple times at night	26 (3.72)	58 (10.5)
Disorganized sleep schedule	13 (1.86)	25 (4.53)
Loss of appetite	23 (3.29)	40 (7.25)
Increased appetite	21 (3.00)	30 (5.43)
Euphoria	13 (1.86)	16 (2.90)
Irritability	78 (11.2)	68 (12.3)
Exaggerated worries	31 (4.43)	55 (9.96)
Sadness	20 (2.86)	70 (12.7)
Nervousness	83 (11.9)	131 (23.7)
Use of alcohol or other drugs	1 (0.14)	9 (1.63)
Aggressiveness	8 (1.14)	20 (3.62)
Child’s behavior, *n* (%)		
Bad or very bad	5 (0.72)	13 (2.36)
Average	96 (13.7)	134 (24.3)
Very good or good	598 (85.6)	405 (73.4)
Presence of ND, *n* (%)	164 (23.5)	―
Video games use, *n* (%)	403 (57.7)	272 (49.3)
**Physical environment during lockdown**		
Length of lockdown (days), *M* (*SD*)	78.8 (5.12)	78.7 (5.29)
City of residence, *n* (%)		
<2000 inhabitants	8 (1.14)	9 (1.63)
2000–10,000 inhabitants	80 (11.4)	61 (11.1)
10,000–50,000 inhabitants	208 (29.8)	264 (47.8)
>50,000 inhabitants	403 (57.7)	215 (38.9)
Comfort Home (range = 2–11), *M* (*SD*)	7.39 (2.28)	6.31 (3.04)
Study Facilities (range = 1–7), *M* (*SD*)	5.44 (1.47)	5.75 (1.40)
**Accompaniment during lockdown**		
People sharing lockdown—parents, *n* (%)		
Both parents	606 (86.7)	474 (85.9)
Single parent	88 (12.6)	75 (13.6)
No parents	5 (0.72)	3 (0.54)
People sharing lockdown—siblings, *n* (%)		
No siblings	183 (26.2)	107 (19.4)
1 sibling	412 (58.9)	289 (52.4)
2 siblings	89 (12.7)	105 (19.0)
≥3 siblings	15 (2.15)	50 (9.06)
Online communication with relatives, *n* (%)		
1 (Not at all)	16 (2.29)	26 (4.71)
2	72 (10.3)	57 (10.3)
3	183 (26.2)	99 (17.9)
4	202 (28.9)	166 (30.1)
5 (Extremely)	226 (32.3)	204 (37.0)
Online communication with friends, *n* (%)		
1 (Not at all)	18 (2.58)	14 (2.54)
2	94 (13.4)	38 (6.88)
3	164 (23.5)	93 (16.8)
4	174 (24.9)	129 (23.4)
5 (Extremely)	249 (35.6)	278 (50.4)
Participation in online classes, *n* (%)		
1 (Never)	44 (6.29)	11 (1.99)
2 (Occasionally)	96 (13.7)	51 (9.24)
3 (Once a week)	197 (28.2)	38 (6.88)
4 (2–3 times a week)	150 (21.5)	108 (19.6)
5 (Every day)	212 (30.3)	344 (62.3)
**COVID-related variables**		
Economic problems, *n* (%)	252 (36.1)	136 (24.6)
Concerned about health problems, *n* (%)		
1 (Not at all)	127 (18.2)	67 (12.1)
2	143 (20.5)	98 (17.8)
3	179 (25.6)	166 (30.1)
4	119 (17.0)	120 (21.7)
5 (Extremely)	131 (18.7)	101 (18.3)
Children diagnosed with COVID-19, *n* (%)	6 (0.85)	9 (1.63)
Relative or friend got COVID-19, *n* (%)	146 (20.9)	161 (29.1)
Relative or friend hospitalized because of COVID-19, *n* (%)	51 (7.29)	63 (11.4)
Relative or friend died because of COVID-19, *n* (%)	14 (2.00)	21 (3.80)

*M*, mean; *SD*, standard deviation; ND, neurodevelopmental disorders.

**Table 2:** Changes in psychological symptoms before and during the COVID-19 lockdown.

	Before Lockdown	During Lockdown		
SDQ Parent (*N* = 699)	*M* (*SD*)	*M* (*SD*)	*p*	Effect Size (*r*)
Emotional symptoms	1.63 (1.85)	2.16 (2.03)	<0.001	0.19
Conduct problems	1.26 (1.52)	1.74 (1.51)	<0.001	0.25
Hyperactivity/inattention	2.99 (2.53)	3.85 (2.67)	<0.001	0.29
Peer problems	1.13 (1.55)	1.65 (1.67)	<0.001	0.24
Prosocial behavior	7.82 (1.88)	7.87 (1.69)	―	
Total difficulties	7.02 (5.57)	9.40 (5.61)	<0.001	0.36
**SDQ adolescent (*N* = 552)**				
Emotional symptoms	2.42 (2.14)	3.29 (2.29)	<0.001	0.26
Conduct problems	1.51 (1.49)	1.92 (1.55)	<0.001	0.16
Hyperactivity/inattention	3.42 (2.44)	4.27 (2.52)	<0.001	0.25
Peer problems	1.27 (1.58)	1.70 (1.67)	<0.001	0.18
Prosocial behavior	7.96 (1.71)	7.81 (1.77)	0.046	0.059
Total difficulties	8.62 (5.59)	11.2 (5.62)	<0.001	0.33

SDQ—Strengths and Difficulties Questionnaire; *M*, mean; *SD*, standard deviation.

**Table 3:** Factors associated with impaired mental health during the COVID-19 lockdown according to parents (*N* = 699).

	*OR* (95% *CI*)	*p*
**Emotional symptoms**		
*Sociodemographic characteristics*		
Educational stage (reference: primary)	1.62 (1.16–2.25)	0.004
*Pre-lockdown situation*		
Exaggerated worries (reference: no)	3.01 (1.28–7.06)	0.011
Waking up multiple times at night (reference: no)	2.87 (1.16–7.11)	0.023
Nervousness (reference: no)	1.67 (1.01–2.75)	0.046
Video games use (reference: no)	0.59 (0.43–0.81)	<0.001
*COVID-related variables*		
Perceived academic delay	1.16 (1.03–1.31)	0.018
**Conduct problems**		
*Pre-lockdown situation*		
Child’s behavior	0.54 (0.42–0.70)	<0.001
*COVID-related variables*		
Following daily routines	0.83 (0.71–0.98)	0.027
**Hyperactivity/inattention**		
*Sociodemographic characteristics*		
Gender (reference: female)	1.41 (1.04–1.92)	0.027
Age	0.94 (0.89–1.00)	0.043
*Accompaniment during lockdown*		
Following virtual learning	0.85 (0.55–0.99)	0.035
**Peer problems**		
*Pre-lockdown situation*		
Sadness (reference: no)	4.16 (1.47–11.8)	0.007
Video games use (reference: no)	0.60 (0.44–0.82)	0.001
*Accompaniment during lockdown*		
Online communication with friends	0.74 (0.64–0.84)	<0.001
**Total difficulties**		
*Pre-lockdown situation*		
Presence of ND (reference: no)	0.52 (0.36–0.74)	<0.001
Video games use (ref. no)	0.65 (0.46–0.90)	0.011

*OR*, odds ratio; *CI*, confidence interval; ND, neurodevelopmental disorders.

**Table 4:** Factors associated with impaired mental health during the COVID-19 lockdown according to adolescents (*N* = 552).

	*OR* (95% *CI*)	*p*
**Emotional symptoms**		
*Pre-lockdown situation*		
Waking up multiple times at night (reference: no)	2.11 (1.14–3.93)	0.018
*COVID-related variables*		
Health concerns	1.18 (1.03–1.36)	0.017
**Conduct problems**		
*COVID-related variables*		
Academic advantage of lockdown	0.81 (0.71–0.93)	0.004
Hyperactivity/inattention		
*Physical environment*		
Study facilities	0.83 (0.74–0.94)	0.005
*COVID-related variables*		
Academic advantage of lockdown	0.79 (0.68–0.91)	0.001
**Peer problems**		
*Pre-lockdown situation*		
Disorganized sleep schedule (reference: no)	3.23 (1.32–7.92)	0.010
*Accompaniment during lockdown*		
Online communication with friends	0.79 (0.68–0.93)	0.005
**Total difficulties**		
*COVID-related variables*		
Economic problems (reference: no)	1.88 (1.21–2.90)	0.005

*OR*, odds ratio; *CI*, confidence interval.

## Data Availability

The datasets generated during and/or analyzed during the current study are available from the corresponding author upon reasonable request.

## Supplementary Materials

The following supporting information can be downloaded at: https://www.mdpi.com/article/10.3390/ijerph19074120/s1, Table S1: Items and response options from the online survey completed by parents of children aged 6 to 17 years during the coronavirus disease 2019 (COVID-19) lockdown; Table S2: Items and response options from the online survey completed by adolescents aged 12 to 17 years during the coronavirus disease 2019 (COVID-19) lockdown; Table S3: Univariate analyses between the items included in the online survey and changes across the significant SDQ subscales according to parents (*N* = 699); Table S4: Univariate analyses between the items included in the online survey and changes across the significant SDQ subscales according to adolescents (*N* = 552).
