# Supplementary material for: Changes in the Mental Health of Children and Adolescents during the COVID-19 Lockdown: Associated Factors and Life Conditions
Source: Int J Environ Res Public Health. 2022 Mar 30;19(7):4120. doi: 10.3390/ijerph19074120 (PMC8998498; doi:10.3390/ijerph19074120)
Supplement: Supplementary file 1 [file ijerph-19-04120-s001.zip › ijerph-1601883-supplementary.pdf]

Supplemental Material for

Changes in the Mental Health of Children and Adolescents during the COVID-19 Lockdown: Associated Factors and Life Conditions

Table S1 Items and response options from the online survey completed by parents of children aged 6 to 17 years during the coronavirus disease 2019 (COVID-19) lockdown

	Question	Response scale
**Sociodemographic characteristics**	Who answers the questionnaire?	Mother, Father, Legal guardian, Others (please describe)
	Child's date of birth	mm/dd/yyyy
	Child's school	Free type
	Child's gender	Male, Female
**Pre-lockdown situation**	How was the family environment before lockdown?	From 1 (very bad) to 5 (very good)
	How was your child's behavior before lockdown?	From 1 (very bad) to 5 (very good)
	Did your child have any of these difficulties before lockdown? Please check as many as necessary	Insomnia, Hypersomnia (sleep more than usual), Waking up multiple times at nigh (at least two), Disorganized sleep schedule, Loss of appetite, Increased appetite, Euphoria, Irritability, Exaggerated worries, Sadness, Nervousness, Use of alcohol or any other drug, Aggressiveness, Others (please describe), None of these
	Does your child have a diagnosis of a neurodevelopmental disorder (e.g., attention-deficit/hyperactivity disorder, learning disorder, autism spectrum disorder)?	No, Yes
	Did your child receive any medication before lockdown?	No, Yes
	Did your child receive any other type of treatment (e.g., psychological, pedagogical, speech therapy) before lockdown?	No, Yes
	Did your child play video games before lockdown?	No, Yes
**Physical environment during lockdown**	City of residence during lockdown	Free type
	Which of these characteristics best describe your child's housing during lockdown? Please check as many as necessary	Apartment, House, Big (> 120 m^2), Medium (90 – 120 m^2), Small (70 – 89 m^2), Very small (40 – 69 m^2), Yard, Garden, Terrace, Balcony, Bright, Others (please describe)
	Which of these facilities have been available to your child during lockdown? Please check as many as necessary	Computer, Internet connection at home, Own cell phone, Tablet, A place to study at home, Own desk, Own bedroom

**Accompaniment during lockdown**	With who has your child lived during lockdown? Please check as many as necessary	Mother (1), Mother (2), Father (1), Father (2), One sibling, Two siblings, Three siblings, Four siblings, Five siblings, Grandfather, Grandmother, Others (please describe), Alone
	How has the family environment been during lockdown?	From 1 (very bad) to 5 (very good)
	Did your family enjoy the time spent together during lockdown?	From 1 (much less than usual) to 5 (much more than usual)
	Has any relative been far from home because of the lockdown?	No, Yes
	During the lockdown period, has your child stayed in touch with relatives who lived apart through online communication systems?	From 1 (not at all) to 5 (extremely)
	During the lockdown period, has your child stayed in touch with friends through online communication systems?	From 1 (not at all) to 5 (extremely)
	Has your child followed online classes with his or her teachers during lockdown?	1 (never), 2 (occasionally), 3 (once a week) 4 (2–3 times a week), 5 (every day)
	Degree of satisfaction with the response given by the school during lockdown from an academic point of view	From 1 to 10
	Have you been able to help your child with his or her homework during lockdown?	From 1 (not at all) to 5 (extremely)
**COVID-related variables**	Have you been concerned about the health of your child or loved ones during lockdown?	From 1 (not at all) to 5 (extremely)
	Has your child contracted COVID-19?	No, Yes
	Has your child been hospitalized for COVID-19?	No, Yes
	How long has your child been hospitalized?	Days
	Has any of your child’s relatives got infected with COVID-19?	No, Yes
	What relationship does your child have with this person?	Mother, Father, Sibling, Grandfather, Grandmother, Aunt, Uncle, Cousin, Other (please describe)
	Has this relative been hospitalized for COVID-19?	No, Yes
	How long has this relative been hospitalized?	Days
	Has any of your child’s relatives died from COVID-19?	No, Yes
	Has any of your child’s close friends got infected with COVID-19?	No, Yes
	Has this friend been hospitalized for COVID-19?	No, Yes
	How long has this friend been hospitalized?	Days
	Has any of your child’s close friends died from COVID-19?	No, Yes
	Has your household income declined due to the pandemic?	No, Yes

	How serious have these economic problems been?	From 1 (mild) to 5 (extremely)
	Has your child maintained his or her daily habits and routines (e.g., hygiene, diet, sleep, study) during lockdown?	From 1 (not at all) to 5 (extremely)
	Has your child practiced physical activity during lockdown?	From 1 (not at all) to 5 (extremely)
	Do you think that your child has taken advantage of lockdown to spend more time on his or her school tasks?	From 1 (not at all) to 5 (extremely)
	Do you think that your child's learning has been delayed by the lockdown?	From 1 (not at all) to 5 (extremely)
	Have your child played video games during lockdown?	From 1 (much less than usual) to 5 (much more than usual)
	Has your child complied with the stay-at-home orders?	From 1 (not at all) to 5 (extremely)
**Mental health during lockdown (Strengths and Difficulties Questionnaire; Goodman, 1997)**	Considerate of other people's feelings	From 0 (not true) to 2 (certainly true)
	Restless, overactive, cannot stay still for long	From 0 (not true) to 2 (certainly true)
	Often complains of headaches, stomach-aches or sickness	From 0 (not true) to 2 (certainly true)
	Shares readily with other children (e.g., toys, treats, pencils)	From 0 (not true) to 2 (certainly true)
	Often has temper tantrums or hot tempers	From 0 (not true) to 2 (certainly true)
	Rather solitary, tends to play alone	From 0 (not true) to 2 (certainly true)
	Generally obedient, usually does what adults request	From 0 (not true) to 2 (certainly true)
	Many worries or often seems worried	From 0 (not true) to 2 (certainly true)
	Helpful if someone is hurt, upset, or feeling ill	From 0 (not true) to 2 (certainly true)
	Constantly fidgeting or squirming	From 0 (not true) to 2 (certainly true)
	Has at least one good friend	From 0 (not true) to 2 (certainly true)
	Often fights with other children or bullies them	From 0 (not true) to 2 (certainly true)
	Often unhappy, downhearted or tearful	From 0 (not true) to 2 (certainly true)
	Generally liked by other children	From 0 (not true) to 2 (certainly true)
	Easily distracted, concentration wanders	From 0 (not true) to 2 (certainly true)
	Nervous or clingy in new situations, easily loses confidence	From 0 (not true) to 2 (certainly true)
	Kind to younger children	From 0 (not true) to 2 (certainly true)
	Often lies or cheats	From 0 (not true) to 2 (certainly true)
	Picked on or bullied by other children	From 0 (not true) to 2 (certainly true)
	Often volunteers to help others (parents, teachers, other children)	From 0 (not true) to 2 (certainly true)
	Thinks things out before acting	From 0 (not true) to 2 (certainly true)
	Steals from home, school or elsewhere	From 0 (not true) to 2 (certainly true)
	Gets on better with adults than with other children	From 0 (not true) to 2 (certainly true)
	Many fears, easily scared	From 0 (not true) to 2 (certainly true)
	Sees tasks through to the end, good attention span	From 0 (not true) to 2 (certainly true)

Table S2 Items and response options from the online survey completed by adolescents aged 12 to 17 years during the coronavirus disease 2019 (COVID-19) lockdown

	**Question**	**Response scale**
**Sociodemographic characteristics**	Date of birth	mm/dd/yyyy
	School	Free type
	Gender	Male, Female
**Pre-lockdown situation**	How was the family environment before lockdown?	From 1 (very bad) to 5 (very good)
	How was your behavior before lockdown?	From 1 (very bad) to 5 (very good)
	Did you have any of these difficulties before lockdown? Please check as many as necessary	Insomnia, Hypersomnia (sleep more than usual), Waking up multiple times at nigh (at least two), Disorganized sleep schedule, Loss of appetite, Increased appetite, Euphoria, Irritability, Exaggerated worries, Sadness, Nervousness, Use of alcohol or any other drug, Aggressiveness, Others (please describe), None of these
	Did you play video games before lockdown?	No, Yes
**Physical environment during lockdown**	City of residence during lockdown	Free type
	Which of these characteristics best describe your housing during lockdown? Please check as many as necessary	Apartment, House, Big (> 120 m^2), Medium (90 – 120 m^2), Small (70 – 89 m^2), Very small (40 – 69 m^2), Yard, Garden, Terrace, Balcony, Bright, Others (please describe)
	Which of these facilities have been available to you during lockdown? Please check as many as necessary	Computer, Internet connection at home, Own cell phone, Tablet, A place to study at home, Own desk, Own bedroom
**Accompaniment during lockdown**	With who have you lived during lockdown? Please check as many as necessary	Mother (1), Mother (2), Father (1), Father (2), One sibling, Two siblings, Three siblings, Four siblings, Five siblings, Grandfather, Grandmother, Others (please describe), Alone
	How has the family environment been during lockdown?	From 1 (very bad) to 5 (very good)
	Did you enjoy the time you spent with your family during lockdown?	From 1 (much less than usual) to 5 (much more than usual)
	Has any relative been far from home because of the lockdown?	No, Yes

	During the lockdown period, have you stayed in touch with relatives who lived apart through online communication systems?	From 1 (never) to 5 (always)
	During the lockdown period, have you stayed in touch with friends through online communication systems?	From 1 (never) to 5 (always)
	Have you followed online classes with your teachers during lockdown?	1 (never), 2 (occasionally), 3 (once a week) 4 (2–3 times a week), 5 (every day)
	Degree of satisfaction with the response given by your school during lockdown from an academic point of view	From 1 to 10
	Have your family helped you with your homework during lockdown?	From 1 (never) to 5 (always)
**COVID-related variables**	Have you been concerned about your health or your relatives' health during lockdown?	From 1 (not at all) to 5 (very concerned)
	Have you contracted COVID-19?	No, Yes
	Have you been hospitalized for COVID-19?	No, Yes
	How long have you been hospitalized?	Days
	Has any of your relatives got infected with COVID-19?	No, Yes
	What relationship do you have with this person?	Mother, Father, Sibling, Grandfather, Grandmother, Aunt, Uncle, Cousin, Other (please describe)
	Has this relative been hospitalized for COVID-19?	No, Yes
	How long has this relative been hospitalized?	Days
	Has any of your relatives died from COVID-19?	No, Yes
	Has any of your close friends got infected with COVID-19?	No, Yes
	Has this friend been hospitalized for COVID-19?	No, Yes
	How long has this friend been hospitalized?	Days
	Has any of your close friends died from COVID-19?	No, Yes
	Has your family had economic problems due to the pandemic?	No, Yes
	How serious have these economic problems been?	From 1 (mild) to 5 (extremely)
	Have you maintained your daily habits and routines (e.g., hygiene, diet, sleep, study) during lockdown?	From 1 (never) to (always)
	Have you practiced physical activity during lockdown?	From 1 (never) to (always)
	Do you think that you have taken advantage of lockdown to spend more time on your school tasks?	From 1 (not at all) to 5 (extremely)
	Do you think that your learning has been delayed by the lockdown?	From 1 (not at all) to 5 (extremely)

	Have you played video games during lockdown?	From 1 (much less than usual) to 5 (much more than usual)
	Have you complied with the stay-at-home orders?	From 1 (not at all) to 5 (extremely)
**Mental health during lockdown (Strengths and Difficulties Questionnaire; Goodman, 1997)**	I try to be nice to other people. I care about their feelings	From 0 (not true) to 2 (certainly true)
	I am restless, I cannot stay still for long	From 0 (not true) to 2 (certainly true)
	I get a lot of headaches, stomach-aches or sickness	From 0 (not true) to 2 (certainly true)
	I usually share with others (e.g., CD's, games, food)	From 0 (not true) to 2 (certainly true)
	I get very angry and often lose my temper	From 0 (not true) to 2 (certainly true)
	I would rather be alone than with people of my age	From 0 (not true) to 2 (certainly true)
	I usually do as I am told	From 0 (not true) to 2 (certainly true)
	I worry a lot	From 0 (not true) to 2 (certainly true)
	I am helpful if someone is hurt, upset or feeling ill	From 0 (not true) to 2 (certainly true)
	I am constantly fidgeting or squirming	From 0 (not true) to 2 (certainly true)
	I have one good friend or more	From 0 (not true) to 2 (certainly true)
	I fight a lot. I can make other people do what I want	From 0 (not true) to 2 (certainly true)
	I am often unhappy, depressed or tearful	From 0 (not true) to 2 (certainly true)
	Other people my age generally like me	From 0 (not true) to 2 (certainly true)
	I am easily distracted, I find it difficult to concentrate	From 0 (not true) to 2 (certainly true)
	I am nervous in new situations. I easily lose confidence	From 0 (not true) to 2 (certainly true)
	I am kind to younger children	From 0 (not true) to 2 (certainly true)
	I am often accused of lying or cheating	From 0 (not true) to 2 (certainly true)
	Other children or young people pick on me or bully me	From 0 (not true) to 2 (certainly true)
	I often offer to help others (parents, teachers, children)	From 0 (not true) to 2 (certainly true)
	I think before I do things	From 0 (not true) to 2 (certainly true)
	I take things that are not mine from home, school or elsewhere	From 0 (not true) to 2 (certainly true)
	I get along better with adults than with people my own age	From 0 (not true) to 2 (certainly true)
	I have many fears, I am easily scared	From 0 (not true) to 2 (certainly true)
	I finish the work I'm doing. My attention is good	From 0 (not true) to 2 (certainly true)

Table S3 Univariate analyses between the items included in the online survey and changes across the significant SDQ subscales according to parents (*N* = 699)

	Emotional symptoms	Conduct problems	Hyperactivity/inattention	Peer problems	Total difficulties
**Sociodemographic characteristics**					
Child's gender	0.004	—	0.012	—	—
Child's age	< 0.001	—	< 0.001	—	—
Educational stage	< 0.001	—	< 0.001	—	—
Type of school	—	—	—	—	—
**Pre-lockdown situation**					
Family environment	—	< 0.001	—	—	—
Child's behavior	—	< 0.001	—	—	—
Insomnia	—	—	—	—	—
Hypersomnia	—	—	—	—	—
Waking up multiple times at night	0.007	—	—	—	—
Disorganized sleep schedule	—	—	—	—	—
Loss of appetite	—	—	—	—	—
Increased appetite	—	—	—	—	—
Euphoria	—	—	—	—	—
Irritability	—	0.005	—	—	—
Exaggerated worries	0.002	—	—	—	—
Sadness	—	—	—	0.012	—
Nervousness	0.001	—	—	—	—
Use of alcohol/drugs	—	—	—	—	—
Aggressiveness	—	—	—	—	—
Presence of neurodevelopmental disorders	—	—	—	—	< 0.001
Medication	—	—	—	—	—
Other treatment	—	—	—	—	—
Video games use	0.001	—	—	0.002	0.006
**Physical environment**					
Comfort Home	—	—	—	—	—
Study Facilities	0.004	—	—	—	—
**Accompaniment during lockdown**					
People sharing lockdown	—	—	—	—	—
Family environment	—	—	—	—	—
Satisfaction with family time spent together	—	—	—	—	—
Relatives living apart	—	—	—	—	—
Online communication with relatives	—	—	—	—	—
Online communication with friends	0.009	—	—	< 0.001	—
Following virtual learning	—	—	< 0.001	—	—
Satisfaction with school response	—	—	—	—	—
Involvement in child's homework	—	—	—	—	—

Table S3 (Continued from previous page)

	Emotional symptoms	Conduct problems	Hyperactivity/inattention	Peer problems	Total difficulties
**COVID-related variables**					
Health concerns during lockdown	—	—	—	—	—
Children diagnosed with COVID-19	—	—	—	—	—
Children hospitalized because of COVID-19	—	—	—	—	—
Duration of child's hospitalization	—	—	—	—	—
Child's relative got COVID-19	—	—	—	—	—
Child's relative hospitalized because of COVID-19	—	—	—	—	—
Duration of child's relative hospitalization	—	—	—	—	—
Child's relative died because of COVID-19	—	—	—	—	—
Child's friend got COVID-19	—	—	—	—	—
Child's friend hospitalized because of COVID-19	—	—	—	—	—
Duration of child's friend hospitalization	—	—	—	—	—
Child's friend died because of COVID-19	—	—	—	—	—
Economic problems during lockdown	—	—	—	—	—
Severity of economic problems	—	—	—	—	—
Following daily routines during lockdown	—	< 0.001	—	—	—
Physical activity during lockdown	—	0.005	—	—	—
Academic advantage of lockdown	—	0.005	—	—	—
Perceived academic delay during lockdown	0.003	0.005	—	—	—
Video games use during lockdown	—	—	—	—	—
Compliance with stay-at-home orders	—	—	—	—	—

SDQ, Strengths and Difficulties Questionnaire.

Significance threshold at $p \leq 0.013$, considering the number of significant SDQ subscales in the parent-reported sample

Table S4 Univariate analyses between the items included in the online survey and changes across the significant SDQ subscales according to adolescents (*N* = 552)

	Emotional symptoms	Conduct problems	Hyperactivity/inattention	Peer problems	Prosocial behavior	Total difficulties
**Sociodemographic characteristics**						
Gender	—	—	—	—	—	—
Age	—	—	—	—	—	—
Educational stage	—	—	—	—	—	—
Type of school	—	—	—	—	—	—
**Pre-lockdown situation**						
Family environment	—	—	—	—	—	—
Behavior	—	—	—	—	—	—
Insomnia	—	—	—	—	—	—
Hypersomnia	—	—	—	—	—	—
Waking up multiple times at night	0.007	—	—	—	—	—
Disorganized sleep schedule	—	—	—	0.008	—	—
Loss of appetite	—	—	—	—	—	—
Increased appetite	—	—	—	—	—	—
Euphoria	—	—	—	—	—	—
Irritability	—	—	—	—	—	—
Exaggerated worries	—	—	—	—	—	—
Sadness	—	—	—	—	—	—
Nervousness	—	—	—	—	—	—
Use of alcohol/drugs	—	—	—	—	—	—
Aggressiveness	—	—	—	—	—	—
Presence of neurodevelopmental disorders	—	—	—	—	—	—
Medication	—	—	—	—	—	—
Other treatment	—	—	—	—	—	—
Video games use	—	—	—	—	—	—
**Physical environment**						
Comfort Home	—	—	—	—	—	—
Study Facilities	—	—	0.001	—	—	—
**Accompaniment during lockdown**						
People sharing lockdown	—	—	—	—	—	—
Family environment	—	—	—	—	—	—
Satisfaction with family time spent together	—	—	—	—	—	—
Relatives living apart	—	—	—	—	—	—
Online communication with relatives	—	—	—	—	—	—
Online communication with friends	—	—	—	0.003	—	—
Following virtual learning	—	—	—	—	—	—
Satisfaction with school response	—	—	—	—	—	—
Parental involvement in homework	—	—	—	—	—	—

Table S4 (Continued from previous page)

	Emotional symptoms	Conduct problems	Hyperactivity/inattention	Peer problems	Prosocial behavior	Total difficulties
**COVID-related variables**						
Health concerns during lockdown	< 0.001	—	—	—	—	—
Diagnosed with COVID-19	—	—	—	—	—	—
Hospitalized because of COVID-19	—	—	—	—	—	—
Duration of hospitalization	—	—	—	—	—	—
Relative got COVID-19	—	—	—	—	—	—
Relative hospitalized because of COVID-19	—	—	—	—	—	—
Duration of relative's hospitalization	—	—	—	—	—	—
Relative died because of COVID-19	—	—	—	—	—	—
Friend got COVID-19	—	—	—	—	—	—
Friend hospitalized because of COVID-19	—	—	—	—	—	—
Duration of friend's hospitalization	—	—	—	—	—	—
Friend died because of COVID-19	—	—	—	—	—	—
Economic problems during lockdown	—	—	—	—	—	0.005
Severity of economic problems	—	—	—	—	—	—
Following daily routines during lockdown	—	—	—	—	—	—
Physical activity during lockdown	—	—	—	—	—	—
Academic advantage of lockdown	—	0.004	< 0.001	—	—	—
Perceived academic delay during lockdown	—	—	0.004	—	—	—
Video games use during lockdown	—	—	—	—	—	—
Compliance with stay-at-home orders	—	—	—	—	—	—

SDQ, Strengths and Difficulties Questionnaire.

Significance threshold at $p \leq 0.010$ considering the number of significant SDQ subscales in the adolescent-reported sample
